# Sequential Smoothing Treatment of Glass Workpieces Cut by Abrasive Water Jet

**DOI:** 10.3390/ma15196894

**Published:** 2022-10-05

**Authors:** Marzena Sutowska, Czesław Łukianowicz, Monika Szada-Borzyszkowska

**Affiliations:** 1 Department of Production Engineering, Faculty of Mechanical Engineering, Koszalin University of Technology, Racławicka 15-17, 75-620 Koszalin, Poland; 2 Department of Engineering of Technical and Information Systems, Faculty of Mechanical Engineering, Koszalin University of Technology, Racławicka 15-17, 75-620 Koszalin, Poland

**Keywords:** sequential smoothing treatment, abrasive water jet machining, surface roughness, soda-lime glass

## Abstract

A centrifugal disc and vibratory finishing machines were applied to improve the surface texture of soda-lime workpieces cut by an abrasive water jet. This innovative method was denoted as sequential smoothing treatment. An experimental study of the effect of the smoothing process conditions on the surface roughness, surface texture and micro roughness of the surface of glass workpieces was conducted. The analysis of the results obtained from experimental research made it possible to determine the optimum conditions for the smoothing process of glass workpieces after abrasive water jet cutting process. The proper selection of the finishing machine, machining media (abrasive chips) and compounds (liquids and powders) made it possible to reduce the surface roughness of areas located in the lower part of the cutting zone from *Sa* = 4.81 μm to *Sa* = 1.9 μm. The experimental results obtained confirmed the validity of using finishing machines to improve the surface quality of the soda-lime glass components. An important problem that requires further research is the increase in productivity and the reduction in machining time.

## 1. Introduction

Abrasive water jet machining is one of the unconventional material shaping methods that may replace other and more traditional machining techniques, which was presented by Llanto et al. [1]. As stated by Krajcarz et al. [2], this is undoubtedly due to the numerous advantages of this machining method. Water jet cutting technology has a number of distinct advantages, such as no thermal distortion, high flexibility, high machining versatility, good surface quality, easy integration with mechanical manipulators, small machining force and minimal burrs, as described by Liu et al. [3]. Abrasive water jets can cut both hard and delicate materials with a wide range of thicknesses with a very low machining force, preventing the destruction of the properties of the target workpiece [4]. This makes the high-pressure water jet a versatile technological tool with an ever-widening range of machining applications, including, among others, mechanical engineering, automotive, aviation and aerospace industries, plastics production and even medicine. A highly concentrated water jet with abrasive grains enables the shaping of a wide range of modern and conventional materials, such as metals and their alloys [5,6,7,8,9], composites [10,11], glass [12], ceramics, natural stone-based materials [13], and it may serve the purpose of regenerating the cutting ability of grinding tools [14].

Abrasive water jet machining consists of shaping materials with a highly concentrated water jet, including abrasive grains, as reported by Natarajan et al. [15]. The locally injected enormous amount of cumulative power (in the form of accelerated abrasive grains) induces effective erosion of the material due to the detachment of its microparticles from the basic mass, as described by Wang and Yang [16]. In this process, the kinetic energy of the abrasive grains is converted into the potential energy of the deformation of the material in the machining zone, as shown by Bitter [17].

The generation of the abrasive water jet is carried out as follows. The water flows from the pump to a water jet orifice (made of ruby, sapphire or diamond) through a high-pressure conduit (a hose). The orifice diameter is between 0.13 and 0.4 mm [18]. In this way, the potential energy of water under high pressure is converted into the kinetic energy of the jet formed. As the jet flows out of the nozzle, it creates a vacuum inside the mixing chamber, which allows the abrasive material to be sucked in. This phenomenon is closely related to the Venturi effect, and it was described by Hashish [19]. As an abrasive, natural abrasives, such as garnet [20] (almandine is the most popular form of garnet used in AWJ technology), Al_2_O_3_ [21], SiC [22] or olivine, are commonly used in the abrasive water jet forming process. The final formation of the jet takes place in a focusing tube, after which the jet is directed at the workpiece. The abrasive water jet thus generated consists of three phases: air (95%), water (ca. 4%) and abrasive particles (1%).

Material removal with a high-pressure water jet is the result of the interaction of the abrasive particles with the workpiece material. The abrasive particles, accelerated by the high-pressure water jet, reach such a high kinetic energy that when they hit the workpiece material, they cause its local deformation and an effective weight loss of the material being machined. This has been described in the literature by Junkar et al. [23].

In addition to the advantages described above, the AWJM method has certain limitations regarding its use. When cutting materials with a high-pressure water jet, an important phenomenon from the perspective of the process quality can be observed, namely a deflection of the jet in the direction opposite to the movement of the cutting head. This means that as the cutting head moves along the workpiece, the outflow of the jet in the area of the lower part of the cutting zone occurs with a certain delay compared to where it enters the material. The distance between the entry and outflow points of the jet is defined by Gostimirovic et al. [24] as the jet lag. The shape that the jet adopts is mapped on the surfaces cut in the form of parallel curved grooves, as reported by Zagórski et al. [25]. The variable nature of the penetration process of the abrasive water jet into the material causes, among other things, disturbances of the surface texture in the lower part of the cutting zone. In this zone, the geometric structure of the surface is machined in the process of abrasive wear (mechanical erosion) and the resulting material loss. The resulting errors reduce the quality of the workpieces cut, as presented in the study by Arola et al. [26].

The quality of surfaces cut by a high-pressure water jet is assessed by determining the dimensional and shape accuracy indices as deviations from the pre-set nominal dimension and by characterising the surface texture parameters. An important role in assessing the technological quality of the cutting process is played by surface roughness parameters considered in 2D and 3D systems. The most commonly used parameters for assessment of the surface texture in 2D, as presented in works by Srivastava et al. [27], Deaconescu and Deaconescu [28], and Phokane et al. [29] and defined in the ISO 4287:1997 standard [30], are the amplitude parameters *Ra*, *Rq*, and *Rz*. The 3D parameters are divided into five groups: amplitude parameters, surface and volume parameters, spatial parameters, hybrid parameters and functional parameters. They are defined in the ISO 25178-2: 2012 Standard [31] and the EN 15178 EUR Report [32]. They are commonly used in the description of surface topography in the manufacturing processes, as demonstrated by Krolczyk et al. [33]. A significant influence of cutting parameters was observed in the case of amplitude parameters. The shape of the topography of the surfaces cut by AWJ also indicates the occurrence of waviness, as presented by Naser et al. [34], the intensity of which increases with the distance from the jet’s entry zone into the material. The set of undulation parameters defined in the ISO 4287: 1997 Standard can be used to quantify this phenomenon.

Abrasive water jet machining comprises several input process parameters, which ultimately determine the efficiency and quality of the machining processes. These parameters are generally categorised as hydraulic, abrasives, cutting and mixing as well as acceleration [35]. The basic process parameters that characterise the cutting of the materials by the abrasive water jet are as follows: water jet pressure (*p*), feed rate (*v_f_*), abrasive flow rate (*m_a_*), water jet orifice diameter (*d_o_*), focusing tube diameter (*d_f_*) and standoff distance (*l*) [36].

An appropriate combination of AWJM input parameters is important to achieve the required machining efficiency and the material’s surface qualities. This has been described in the literature by Krenicky et al. [5]. The authors presented an interesting study on the influence of the cutting process parameters of the water jet cutting of HardoxTM steels on the surface quality. On the basis of the results obtained, they focused on the influence of the processing conditions on the arithmetic mean deviation of the roughness profile (*Ra*) and the height of the profile (*Rz*); mathematical models of the cutting process were developed, which can be used to predict the quality of the surfaces machined by a high-pressure water jet.

A Xiong et al. study demonstrated interesting results [37]. At first, through an analysis of preliminary experimental data, the authors explored an optimal variable standoff distance of AWJ cutting titanium alloy under single cutting. Then, the advantages of reverse cutting with a variable standoff distance strategy were verified. Finally, the authors studied the effects of this strategy on the cutting kerf taper and the surface quality of the cross-section.

Gostimirovic et al. [24] presented an interesting method of optimisation of AWJM process parameters in order to reduce the jet lag effect. The authors used evolutionary algorithms and genetic programming to determine the model of the trajectory curvature.

So far, it has been possible to obtain a roughness of abrasive water jet machined surfaces in the range *Ra* = 2.12 ÷ 22.50 μm (Table 1). Improving the surface quality of the workpieces obtained after the water jet cutting process is an area that has scope for further research because it is under-explored or outdated in the research literature.

Glass has many different applications in optics, fibre optic technology and medicine but also as a construction material in construction, chemical industry, lighting technology, industrial design, etc. The surfaces of glass objects are smoothed most often due to the desire to obtain the appropriate optical, chemical and aesthetic properties. In the technology of optical elements, advanced methods of smoothing the glass surface, such as polishing, lapping and many others, are used. However, in the case of lower requirements regarding the surface texture, methods such as grinding [42] and abrasive suspension jet processing are used. In this paper, to smooth the glass surface after cutting by AWJ, we propose a method involving the use of a centrifugal disc finishing technology, and then, vibration finishing technology. This method is referred to in this article as sequential smoothing treatment.

## 2. Methodology of Experimental Studies

### 2.1. Main Goal

The primary goal of this paper is to present a new method for improving the surface quality of glass workpieces cut by the abrasive water jet. The method described in this paper consists of the use of centrifugal disc and vibratory finishing machines, machining tools (abrasive chips) and machining aids (liquids and powders) for sequential smoothing operations on the surface of workpieces cut with an abrasive water jet. The main research problem in the paper was to determine the sequence of the individual operations, taking into account the type of the finishing machine, the type, shape and size of the chips and the correct selection of machining aids. The innovation of the research described in the article lies in the fact that, although the high surface quality of products manufactured is very important from the manufacturer’s perspective, improving the surface texture after the abrasive water jet machining process is under-explored. In the following sections, details related to the conditions in which the experimental studies were carried out, as well as the results of the experiments, including their analyses, are provided.

### 2.2. Characteristics of the Samples

In this study, soda-lime glass was investigated. The material thickness applied within this study was 15 mm. The choice of this type of material was made deliberately, and it was due to its high susceptibility to hydro-jetting erosion. In addition, soda-lime glass is a brittle material and one that is very difficult to machine using conventional machining techniques.

**Figure 1:**
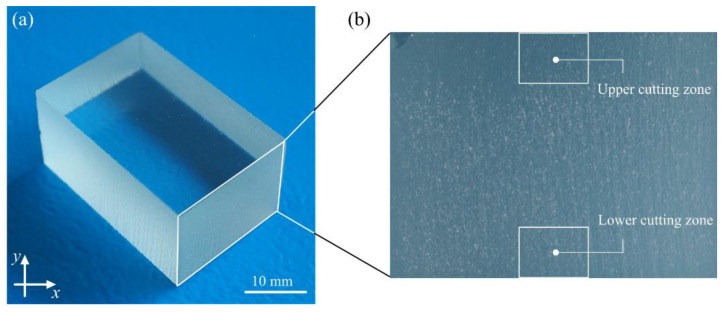
Glass workpiece after abrasive water jet cutting process: (**a**) general view of the glass workpiece; (**b**) a section of the glass workpiece with the upper and lower cutting zone marked.

Soda-lime glass is an amorphous body, one that is produced as a result of supercooling molten raw minerals and other inorganic substances without any crystallisation of the ingredients. This relatively inexpensive and widely available glass is a base material for most types of glass. Its chemical composition and physical properties are detailed in Table 2. The general view of the example glass workpiece used in the experimental studies is presented in Figure 1.

### 2.3. Conditions and Course of the AWJ Process

A high-precision AWJ system, i.e., JetMachining^®^ Center type 55100 (OMAX Corp., Kent, WA, USA), was used to cut soda-lime glass workpieces to 20 × 30 × 15 mm. The AWJ cutting parameters are shown in Table 3. The machine specifications are detailed in Table 4.

### 2.4. Conditions and Course of Sequential Smoothing Machining

Two finishing machines were used to smooth the surface of glass workpieces after the abrasive water jet cutting process: an EC6 type disc finishing machine (Avalon Machines Ltd., Nowa Wieś Lęborska, Poland) and a WE10 type vibratory finishing machine (Avalon Machines Ltd.). The first stage of the smoothing process of the glass workpieces machined with an abrasive water jet was carried out using an EC6 type disc finishing machine. For the tests, 4 kg of T pyramid-shaped green plastic abrasive chips (polyester resin) (marked as 02PP10 type), 3.5 L of H_2_O and 40 mL of ASP-R fluid were used. Avalon Machines Ltd. is a distributor of the 02PP10 chips and ASP-R fluid. During the treatment process, the rotor speed was 295 rpm. The finishing time for one glass workpiece was 3 h.

The body of the EC6 type disc finishing machine is a welded structure created from square profiles, where the working unit is mounted, driven by an electric motor with a belt drive. A separation system is mounted under the working unit. A control panel is mounted next to the working unit to control the technological process. The specifications of the EC6 type disc finishing machine are further detailed in Table 5.

The subsequent steps in the process of smoothing abrasive water jet of glass workpieces were carried out in a WE10 type vibratory finishing machine. For the tests, 10 kg of CMG 3 white porcelain chips and three types of powder were used: GP20, Al_2_O_3_ 800, CeO_2_M_2_. Avalon Machines Ltd. is a distributor of the CMG chips and powders (such as GP20, Al_2_O_3_ 800 and CeO_2_M_2_). 

The base of the WE10 type vibratory finishing machine is a welded structure covered with a laminate, where the working chamber is mounted. The working chamber is covered with polyurethane. The chamber is set into vibratory motion by an electric motor. The split chamber is equipped with a water drain sifter; the water drain system terminates with a valve. A control panel is attached to the WE10 vibratory finishing machine to control the process. The specifications of the WE10 type vibratory finishing machine are further detailed in Table 6.

Table 7 includes the designations of the glass workpieces, including the smoothing process conditions. For the A workpiece, no information on the smoothing process conditions is given because this workpiece is machined with the abrasive water jet cutting process only (for reference purposes).

### 2.5. Characteristics of Measurement Systems and Course of Measurement Process

The geometrical structure of the surface of glass workpieces smoothed in centrifugal disc and vibratory finishing machines was measured by one of the advanced optical methods based on optical profilometry. In carrying out the measurements, a CLI 2000 Talysurf multisensory optical profilometer (Taylor-Hobson, Leicester, UK) was used. The general characteristics of the measurement system used in the experimental studies are presented in Table 8.

The measurement of the surface texture was carried out using a LK-031 type laser sensor (Keyence Corp., Osaka, Japan) installed in the measuring head of the CLI 2000 Talysurf. The measurements of the surface texture were taken in the lower part of the cutting zone. An area with dimensions (*x*, *y* axis) 4.8 × 4.8 mm was measured on each glass workpiece. The number of profiles (*y* axis) was 321. The distance between the profiles (*y* axis) was 15 μm. The number of profile points (*x* axis) was 2401. The distance between the profile points (*x* axis) was 2 μm. The measuring time was 4020 s.

A test stand was used to acquire images of the angular intensity distribution of scattered light. It consisted of a CPS 182 semiconductor laser (Thorlabs, Inc., Newton, MA, USA) with 4.5 mW power, emitting visible radiation with a wavelength λ = 635 nm (red). The laser beam was directed at an incidence angle of 80°, and it illuminated a selected area of a glass workpiece at the bottom of the cutting zone. Reflected and scattered by the surface workpieces, the beam fell on the observation plane, creating an image of the angular intensity distribution of the light scattered on the plane. A 300 × 300 mm opaque screen with a scale for an initial estimation of the dimensions of the resulting image was used as the observation plane. The acquisition of the images of the angular distribution of scattered light intensity was carried out at the bottom of the cutting zone of the glass workpiece. Each time, image registration was performed for a selected section of the zone, measuring 10 × 10 mm. The registration was carried out with a colour camera with a 16 Mp sensor. The following acquisition parameters were used: exposure time = 1/30 s (an electronic shutter was used), image resolution = 2280 × 1080 pixels, recording format = *.bmp. The camera images were transferred to a computer using a USB, where the recorded measurement data were archived. The recorded set of images of angular scattered light intensity distribution was processed and analysed using the Image-Pro^®^Plus 5.1 software (Media Cybernetics, Inc., Rockville, MD, USA).

## 3. Results and Discussion

The analysis of the experimental results was divided into the following stages:

1. The impact of the smoothing process conditions on the surface texture of glass workpieces, carried out on the basis of the calculated values of the roughness parameters (Section 3.1).
2. The impact of the smoothing process conditions on the surface texture of glass workpieces using surface microtopography measured using an optical method (Section 3.2).
3. The impact of the smoothing process conditions on the micro-roughness of the surface of the glass workpieces, carried out on the basis of an analysis of the angular distribution of the intensity of scattered light (Section 3.3).

### 3.1. Surface Roughness

**Figure 2:**
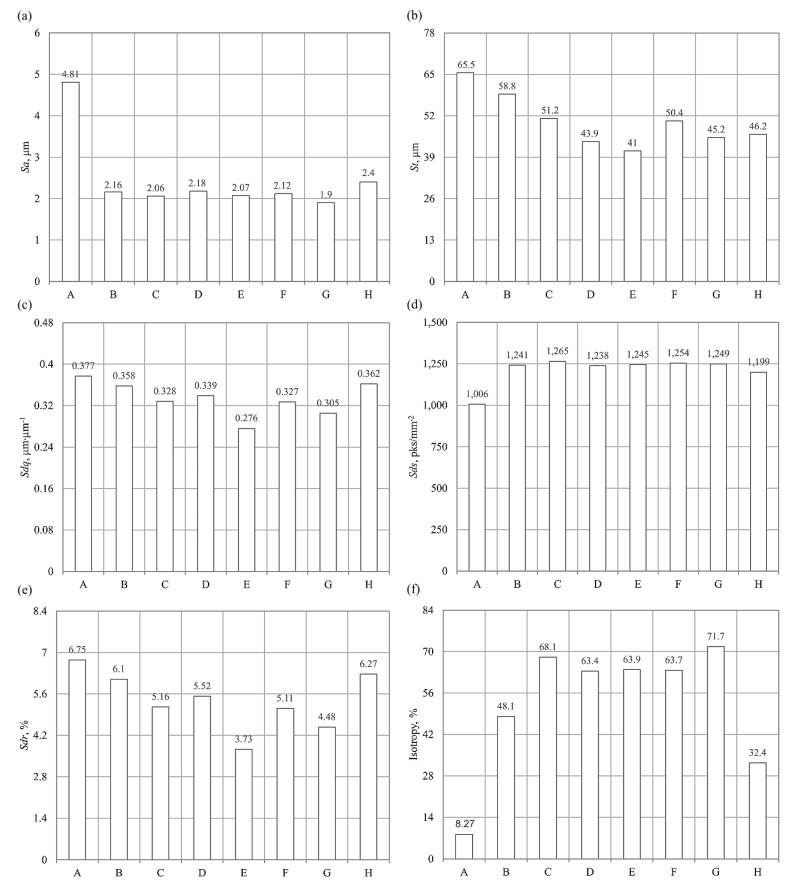
Collection of the selected results of experimental studies in graphical form presenting the calculated values of selected surface texture parameters using TalyMap Silver 4.1.2 software (Digital Surf, Besançon, France): (**a**) *Sa*, (**b**) *St*, (**c**) *Sdq*, (**d**) *Ssc*, (**e**) *Sdr*, (**f**) isotropy.

The relationship between the conditions of the smoothing process of the glass workpieces and the arithmetic mean deviation of the surface *Sa* is presented in Figure 2a. When analysing the graph, it can be seen that the application of the three-stage smoothing process (workpiece G) reduced the value of the *Sa* amplitude (surface) parameter by an average of 60% in the areas located in the lower part of the cutting zone. At the same time, extending the treatment time with GP 20 powder beyond 24 h (workpieces D, E and F) did not improve the surface quality of the workpieces. For example, when the machining time was extended from 24 to 48 h and then to 72 and 96 h, the arithmetic mean deviation of the surface increased by 0.12 μm, 0.1 μm and 0.6 μm, respectively.

The impact of the smoothing process conditions of the glass workpieces on the total height of the surface (*St*), presented in a graphic form (Figure 2b), indicated the existence of significant correlations. The use of resin chips in the process (workpiece B) reduced the value of the *St* parameter by approximately 10%. In addition, it can be seen that the use of the GP20 powder in the subsequent stage of the smoothing process (workpiece C) reduced the surface roughness expressed by the *St* parameter by approximately 22%. Furthermore, the use of a three-stage smoothing process with the Al_2_O_3_ 800 powder (workpiece G) reduced the value of the *St* parameter by more than 30%. On the other hand, the lowest value of the total height of the surface *St* = 41 m was observed when the treatment time was extended to 72 h in the two-stage smoothing process with the GP20 powder (workpiece E).

Figure 2c shows the research results obtained for the root-mean-square slope of the surface (*Sdq*) hybrid parameter. The results indicate that carrying out the smoothing process in finishing machines has an effect on reducing the slope of irregularity of the surface of workpieces. The single-step smoothing process, with the ASP-R fluid, resulted in a reduction in the root-mean-square slope of the surface to 0.358 μm⋅μm^−1^. The smallest value of the *Sdq* parameter (0.276 μm⋅μm^−1^) was obtained in the case of the two-step smoothing process with the GP20 powder (workpiece E). On the other hand, carrying out a three-stage (workpiece G) and four-stage (workpiece H) smoothing process of glass workpieces reduced the values of the root-mean-square slope of the surface to 0.305 μm⋅μm^−1^ and 0.362 μm⋅μm^−1^, respectively.

The research results related to the impact of the smoothing process conditions on the density of summits of the surface (*Sds*) are shown in Figure 2d. When analysing the graph, it can be concluded that carrying out the process in finishing machines increased the value of the (*Sds*) parameter. The measurement results also indicate that the areal (surface) parameter analysed reached its maximum value in the case of the two-stage smoothing process (workpiece C), *Sds* = 1265 μm^2^, while its minimum value was observed in the four-stage smoothing process (workpiece H), *Sds* = 1199 μm^2^.

Figure 2e shows the experimental results of the impact of the glass workpiece smoothing process conditions on the interfacial area ratio (*Sdr*) developed. When analysing the graph, it can be noted that carrying out the glass workpiece smoothing process reduced the value of the hybrid (surface) *Sdr* parameter for the single-stage process by ca. 10% (workpiece B), for the two-stage process (workpiece C) by ca. 24%, for the three-stage process (workpiece G) by ca. 34% and for the four-stage process (workpiece H) by ca. 7%.

The results of the study of the impact of the conditions of the smoothing process on isotropy, presented in a graphic form (Figure 2f), indicate the existence of significant correlations. The results of an analysis of the symmetry of the surface texture of glass workpieces make it possible to conclude that the surface is characterised by the highest isotropy (71.7%), which was subjected to the smoothing process with the Al_2_O_3_ 800 powder (workpiece G). At the same time, the use of the four-stage smoothing process resulted in an increase in isotropy to merely 32.4%.

When analysing the experimental results, it can be concluded that the use of sequential smoothing treatment results in a decrease in the surface roughness of glass material after the abrasive water jet cutting process. The use of the three-stage smoothing process with the Al_2_O_3_ 800 powder (workpiece G) resulted in a decrease in the values of the geometrical structure parameters of the surface, such as *Sa*, *St*, *Sdq* and *Sdr*. The most important of these parameters, (*Sa*), underwent favourable changes, even reaching as much as over 60% of the value. In addition, what also needs to be noted is a positive effect of the process of smoothing glass workpieces in finishing machines on an increased density of the summits of the surface (*Sds*) and isotropy.

### 3.2. Surface Texture

**Figure 3:**
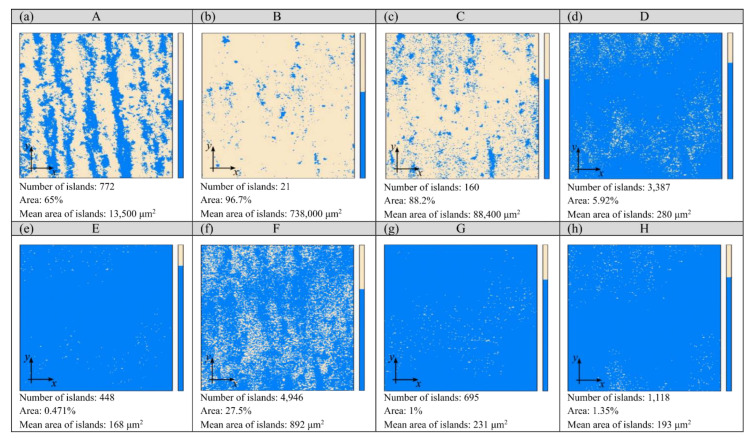
Results of an analysis of islands (number and area of islands) isolated on glass workpieces (marked with uppercase letters A–H) under eight diverse smoothing process conditions (**a**–**h**).

**Figure 4:**
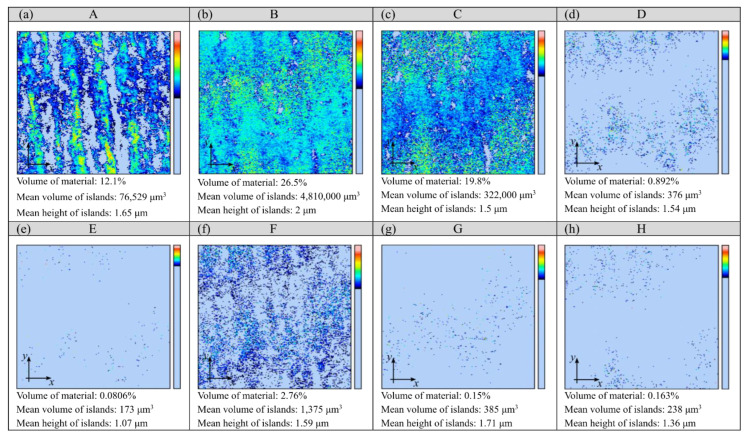
Results of an analysis of islands (volume and height of islands) isolated on glass workpieces (marked with uppercase letters A–H) under eight diverse smoothing process conditions (**a**–**h**).

The smoothing process results in reducing the surface roughness of the workpieces. The magnitude of these changes depends on the conditions adopted; it can be observed on the surface texture. The analysis of the islands made it possible to isolate the surface irregularities of the glass workpieces that were located above the cut-off plane defined at 35 mm from the lowest point of the microtopography. The results of this analysis are shown in Figure 3 and Figure 4. The surface of the glass workpiece subjected to a single-stage smoothing process with resin chips (workpiece B) was characterised by the highest area (96.7%) and volume (26.5%) fraction of the islands among the workpieces evaluated (A ÷ H). In the case of the other workpieces, the values of the parameters describing the islands decreased as a result of extending the treatment time (workpieces C, D, E and F) or carrying out a multi-stage smoothing process (workpieces G and H).

During the machining process in the EC6 type disc finishing machine, the shape of the rotor allowed for the spiral movement of the workpieces and working medium inside the working chamber. The abrasive chips moving in a spiral stream resulted in an intensive abrasive treatment of the glass samples placed in the batch. The use of the ASP-R fluid and 02PP10 chips in the smoothing process resulted in a reduction in the height of the roughness of the machining marks (striation), which translated into an increase in the mean area and the mean volume of the islands (workpiece B). During the machining process in the WE10 type vibratory finishing machine, the working medium inside the working chamber performed a spiral movement, and the soda-lime glass workpiece within it was subjected to a vibration-abrasive treatment. Extending the treatment time to 72 h with the GP20 powder resulted in a flattened area at 35 μm from the lowest microtopography point (workpiece E). The large number of islands with a small area (0.471%) and volume (0.081%) fraction resulted in a small mean value of their mean surface of 168 μm^2^ (Figure 3) and the smallest mean volume of 173 μm^3^ (Figure 4). Such values of the parameters of the islands indicate the presence of numerous yet small micro-areas on the surface of the glass workpiece, which may correspond to the fragments of flattened machining marks.

The island parameters determined in Figure 3 and Figure 4 indicate that the three-stage (workpiece G) and four-stage (workpiece H) smoothing process had a flattening effect on the machining marks, resulting in an increased number of islands. This positive effect was also evident in the case of the area and volume fraction of the islands, the mean surface of the islands and the mean volume of the islands. The area fraction of the islands, when using the three-stage smoothing process with the Al_2_O_3_ 800 powder (Figure 3g) and the four-stage smoothing process with the CeO_2_M_2_ powder (Figure 3h), was approximately 87% lower in comparison to the two-stage smoothing process with the GP20 powder (Figure 3c). The values of the mean surface of the islands obtained for the G and H workpieces were approximately 99% smaller in relation to the C workpiece. The change in the mean volume of the islands was analogous. Carrying out the three- and four-stage smoothing processes reduced the values of this parameter by approximately 99% (Figure 4g,h), respectively, compared to the value determined for the two-stage smoothing process (Figure 4c). All of this demonstrates a significant impact of the smoothing process conditions on the surface texture of the glass workpieces.

### 3.3. Micro-Roughness

**Figure 5:**
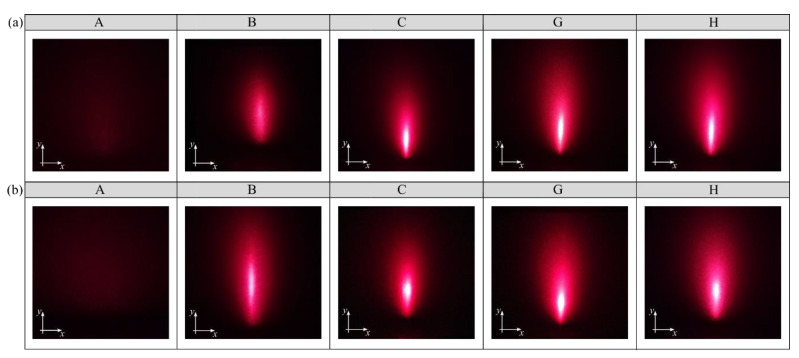
Images of the angular distribution of scattered light intensity obtained from the reflection of a laser light beam with a wavelength λ = 635 nm directed: (**a**) perpendicular; (**b**) parallel at an incidence angle of 80° to machining marks (striation). A-H are identification marks of glass workpieces.

Figure 5 shows examples of the images of angular distribution of scattered light intensity obtained when illuminating the glass workpieces assessed perpendicular and parallel to the machining marks (striation). The local light intensity recorded for selected fragments of the glass workpieces varies with the characteristics of their surface texture. This depends largely on the processing conditions adopted.

**Figure 6:**
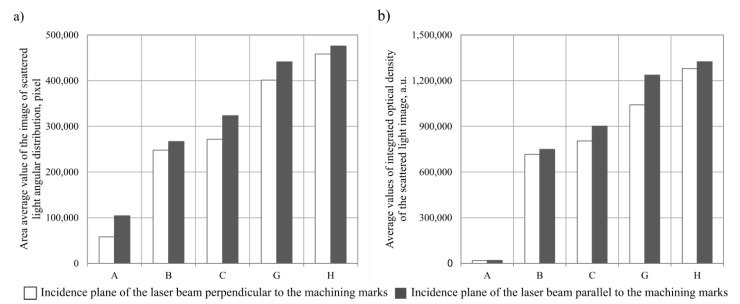
Mean values of the area (bright areas) of scattered light images (**a**) and the total light intensity (bright areas) of scattered light images (**b**) obtained for selected glass workpieces.

Figure 6 presents the results of analyses obtained with the Image-Pro^®^Plus 5.1 software. The results in the form of bar charts were compiled for two parameters of the images evaluated: the area of the scattered light image *An* [43], calculated according to Equation (1), and the total light intensity of the scattered light angular distribution image *I* [43], calculated according to Equation (2).

(1) $$An=\overset{N-1\;\;}{\underset{i=0}{\sum }}\overset{N-1}{\underset{j=0}{\sum }}I\left(i,j\right)$$

where

*i*, *j*—coordinates of the corresponding elements of the images: *i*—row number, *j*—column number;

*I(i*, *j)*—intensity;

(2) $$I_{}=\underset{i,j\in An}{\sum }I\left(i,j\right)$$

where

*i*, *j*—coordinates of the corresponding elements of the images: *i*—row number, *j*—column number;

*I(i*, *j)*—intensity;

*An*—image area of the angular distribution of scattered light intensity.

Due to a relatively large number of the results obtained, the values of the parameters (*An*, *I_Σ_*) were averaged at the level of a single measurement area, consisting of eight thousand four hundred and fifty-two measurement points.

Figure 6a shows the results obtained for the averaged values of the scattered light image areas *An*. The values recorded depended on how the surface of the glass workpiece was illuminated. Higher values were recorded when the incidence plane of the laser light beam was parallel to the machining marks (*An_avg_*_(__∥__)_ = 322,377 pixels). There were more than 10% higher values obtained when the incidence plane of the laser light beam was perpendicular to the machining marks (*An_avg_*_(__⟂__)_ = 287,455 pixels). An analysis of the scattered light image area values leads to the conclusion that the highest values were recorded for the H glass workpiece (*An_avg_* = 475,865 pixels). For the B, C and G glass workpieces, the values recorded were higher (155%, 210% and 322%, respectively) in relation to the A glass workpiece (acting as a reference). Overall, it can be concluded that the smoothing treatment in the finishing machines resulted in approximately 261% higher *An* values than for the A glass workpiece, on average.

An analysis of the total light intensity of the angular scattered light intensity distribution images (Figure 6b) leads to a conclusion that the values of this parameter (irrespective of the glass workpiece assessed) followed a similar trend to the values of the average area of the scattered light images. Here, too, the illumination of the surface in parallel to the machining marks proved to be more beneficial. The mean value of the light intensity of the A glass workpiece (acting as a reference) recorded for this mode of illumination was *I_Σ_* *_avg_*_(__∥__)_ = 19,582 a.u., while for the illumination perpendicular to the machining marks, it was *I_Σ_* *_avg_*_(__∥__)_ = 18,625 a.u. The highest mean value of the total light intensity of the angular scattered light intensity distribution images was obtained for the H glass workpiece (*I_Σ__avg_* = 1,324,398 a.u.). The remaining values obtained were smaller, and these were as follows: B glass workpiece (*I_Σ__avg_* = 748,853 a.u.), C glass workpiece (*I_Σ__avg_* = 901,313 a.u.), G glass workpiece (*I_Σ__avg_* = 1,236,642 a.u.). The percentage increase in *I_Σ__avg_* for the G and H glass workpieces in relation to the A glass workpiece (acting as a reference) averaged 6214% and 6663%, respectively.

The values obtained for both parameters correlated with a visual analysis of the images of the angular distribution of scattered light intensity lead to a conclusion that there is a clear difference between the surfaces of the glass workpieces analysed (A, B, C, G and H). The surface of the A workpiece is characterised by the significantly lower intensity of scattered light compared to the areas of the G and H workpieces. The surface image of the A workpiece (acting as a reference) is characterised by a low intensity of scattered light due to its partial refraction and absorption. This means that the most favourable conditions of the smoothing process, from the perspective of the experimental research, were in the case of the treatment of the G and H workpieces. The use of the three- and four-stage smoothing processes resulted in a significant flattening of the machining marks on the surfaces of the glass workpieces.

## 4. Conclusions

The objective of this paper was to present a new method of improving the surface quality of glass workpieces after an abrasive water jet cutting process (sequential smoothing treatment). The main research problem was solved by conducting an experimental study on the impact of the smoothing process conditions on the surface roughness, the surface texture and the micro-roughness of the surface of glass workpieces.

The results obtained of the measurements and analyses lead to the following conclusions:

1. The three-stage treatment process with the Al2O3 800 powder resulted in the biggest decrease in the arithmetic mean deviation of the surface Sa, reaching an over 60% lower value than the initial one.
2. The greatest flattening of the machining marks was obtained on the surface of the glass workpiece marked with E, which can be explained by extending the machining time to 72 h. A large number of islands with a small area fraction (0.471%) and volume fraction (0.081%) resulted in a small value of their average surface area, amounting to 168 mm^2^, and the smallest average island volume, amounting to 173 mm^3^. Similar results were obtained by analysing the values of the island parameters determined for the three- and four-stage smoothing process.
3. The visual analysis of the angular scattered light intensity distribution images lead to the conclusion that the area of the scattered light image An of the G and H glass workpieces was higher (322% and 355%, respectively) in relation to the reference A glass workpiece. At the same time, a percentage increase in the values of the *I_Σ__avg_* parameter for these workpieces in relation to the A glass workpiece averaged 6214% and 6663%, respectively. It should therefore be assumed that the most favourable conditions for the smoothing process were established for the treatment of the G and H glass workpieces.
4. Taking into account the machining time and the results obtained in the tests on the impact of the smoothing process conditions on the surface roughness, the surface texture and the micro-roughness of the surface of glass workpieces, it can be concluded that the most favourable conditions of the process are to be considered as those described in the article for the G glass workpiece.
5. The experimental results presented in this paper do not exhaust all the issues related to the problem of sequential smoothing treatment, especially the aspects of the surface quality and process time. It is therefore necessary to continue research work in this field. In this situation, it seems reasonable to determine the optimum conditions of the smoothing process for other types of materials, e.g., titanium alloys and composites, which will differ from those applied to soda-lime glass.
6. Reductions in machining time can be achieved by positioning the workpiece in the finishing chamber in an area coinciding with a high kinetic energy potential of the stream. Identifying this area can be achieved by measuring the force or acoustic emission signals.

## Figures and Tables

**Table 1:** Surface roughness studies on AWJM.

Authors	Work Material	Thickness, mm	Variable Process Parameters	Ra, μm
Krenicky et al. [5]	Hardox Steel	6, 10, 15, 40	*v_f_*, *p*, *m_a_*	2.27–7.93
Bañon et al. [10]	Composite materials	1.53	*v_f_*, *p*, *m_a_*	5.21–6.95
Sutowska et al. [12]	Soda-lime glass	8	*r*	2.12–4.99
Abdullah et al. [14]	Marble	20	*v_f_*, *m_a_*, *l*	4.50–8.10
Jegaraj et al. [18]	Aluminium	10	*p*, *m_a_*, *d_o_*, *d_f_*	2.50–22.50
Zagórski et al. [25]	Magnesium alloy	15	*v_f_*, *m_a_*,	3.00–4.00
Akkurt [38]	Brass	5, 10, 15, 20	*v_f_*	2.22–3.47
Arola et al. [39]	Pure titanium	6.4	*p*	3.20–15.50
Boud et al. [40]	Brass		*v_f_*	4.47–7.17
Li et al. [41]	CFRP/Ti6Al4V stacks	3.6	*v_f_*, *p*, *l*	3.04–7.98

**Table 2:** General characteristics of soda-lime glass.

**Chemical Composition**							
SiO_2_, %	Na_2_O, %	CaO, %	MgO, %	Al_2_O_3_, %	K_2_O, %	SO_2_, %	Fe_2_0_3_, %
72.60	13.90	8.40	3.90	1.10	0.60	0.20	0.11
**Strength**							
Flexural				Compressive			
Annealed, MPa		Toughened, MPa		Heat-strengthened, MPa	Annealed, MPa	Toughened, MPa	Heat-strengthened, MPa
41		165		83	19	77	39
**Physical properties**							
Density, kg/m^3 (1)^		Modulus of elasticity, GPa		Mohs hardness, –	Poisson’s ratio, –	Shear modulus, GPa	Coeff. of thermal stress, MPa/°C
2500		72		5–6	0.23	30	0.62
Thermal conductivity, W/m·K		Softening point, °C		Annealing point, °C	Specific heat, kJ/kg·K	Coeff. of linear expansion, °C	Index of refraction, – ^(2)^
0.937		715		548	0.88	8.3·10^−6^	1.5

^(1)^ In temperature 18 °C, ^(2)^ in visible wavelength range *λ* = 380–780 nm.

**Table 3:** Process parameters.

Parameters	Values
Traverse speed, mm/min	174.05
Water jet pressure, MPa	124.11
Abrasive feed rate, kg/min	0.363
Water jet orifice diameter, mm	0.38
Focusing tube diameter, mm	0.76
Standoff distance, mm	1.5

**Table 4:** JetMachining^®^ Center type 55100 specifications.

Parameters	Range
Max pressure, MPa	385
Max water flow rate, dm^3^/min	4.9
Max traverse speed, mm/min	4572
Table size (L × W), mm	3200 × 1650
XY cutting envelope, mm	2540 × 1397
Z-axis travel, mm	205

**Table 5:** Specifications of EC6 type disc finishing machine.

Parameters	Range
Power supply, V	230
Power, kW	0.3
Dimensions (L × W × H), mm	525 × 486 × 702
Weight, kg	33
Working chamber capacity, L	6
Working chamber inside diameter, mm	210

**Table 6:** Specifications of WE10 type vibratory finishing machine.

Parameters	Range
Power supply, V	230
Power, kW	0.14
Dimensions (L × W × H), mm	440 × 380 × 440
Weight, kg	30
Working chamber capacity, L	10
Working chamber inside diameter, mm	310

**Table 7:** Smoothing process conditions.

Glass Workpiece	Machining Stage	Machine Type	Working Medium	Rotational Speed, rpm	Process Time, Hours
A	-	-	-	-	-
B	1	EC6	3.5 L H_2_O + 40 mL ASP-R + 02PP10	295	3
C	1	EC6	3.5 L H_2_O + 40 mL ASP-R + 02PP10	295	3
	2	WE10	500 mL H_2_O + 120 g GP20 + CMG ϕ 3 mm	2800	24
D	1	EC6	3.5 L H_2_O + 40 mL ASP-R + 02PP10	295	3
	2	WE10	500 mL H_2_O + 120 g GP20 + CMG ϕ 3 mm	2800	48
E	1	EC6	3.5 L H_2_O + 40 mL ASP-R + 02PP10	295	3
	2	WE10	500 mL H_2_O + 120 g GP20 + CMG ϕ 3 mm	2800	72
F	1	EC6	3.5 L H_2_O + 40 mL ASP-R + 02PP10	295	3
	2	WE10	500 mL H_2_O + 120 g GP20 + CMG ϕ 3 mm	2800	96
G	1	EC6	3.5 L H_2_O + 40 mL ASP-R + 02PP10	295	3
	2	WE10	500 mL H_2_O + 120g GP20 + CMG ϕ 3 mm	2800	24
	3	WE10	500 mL H_2_O + 50 g Al_2_O_3_ 800 + CMG ϕ 3 mm	2500	24
H	1	EC6	3.5 L H_2_O + 40 mL ASP-R + 02PP10	295	3
	2	WE10	500 mL H_2_O + 120 g GP20 + CMG ϕ 3 mm	2800	24
	3	WE10	500 mL H_2_O + 50 g Al_2_O_3_ 800 + CMG ϕ 3 mm	2500	24
	4	WE10	500 mL H_2_O + 50 g CeO_2_M_2_ + CMG ϕ 3 mm	2400	12

**Table 8:** Characteristics of measurement system used in experimental studies [12].

Instrument Type	Model	Producer	Configuration and Features
Multisensory optical profilometer	CLI2000	Taylor-Hobson (Leicester, UK)	Components: laser triangulation sensor LK-031 (Keyence Corp., Osaka, Japan) Features (sensor): scanning frequency: 2000 Hz, measuring range: 10 mm, resolution: 1 μm (vertical), 30 µm (lateral), measuring slope: 40°, speed: 30 mm/s Features (instrument): measuring capacity: 200 × 200 × 200 mm, axis traverse length: 200 mm, axis resolution: 0.5 μm, dimensions: 800 × 800 × 800 mm, measuring speed: 0.5, 1, 5, 10, 15 and 30 mm/s, positioning speed: 30 mm/s
			Software: Talyscan CLI 2000 2.6.1 + TalyMap Silver 4.1.2 (Digital Surf, Besançon, France)

## References

[1] Llanto J.M., Tolouei-Rad M., Vafadar A., Aamir M. (2021). Impacts of Traverse Speed and Material Thickness on Abrasive Waterjet Contour Cutting of Austenitic Stainless Steel AISI 304L. Appl. Sci..

[2] Krajcarz D., Bańkowski D., Młynarczyk P. (2017). The effect of traverse speed on kerf width in AWJ cutting of ceramic tiles. Procedia Eng..

[3] Liu X., Liang Z., Wen G., Yuan X. (2019). Waterjet machining and research developments: A review. Int. J. Adv. Manuf. Technol..

[4] Sureban R., Kulkarni V.N., Gaitonde V. (2019). Modern optimization techniques for advanced machining processes–A review. Mater. Today Proc..

[5] Krenicky T., Servatka M., Gaspar S., Mascenik J. (2020). Abrasive Water Jet Cutting of Hardox Steels—Quality Investigation. Processes.

[6] Dixit N., Sharma V., Kumar P. (2021). Research trends in abrasive flow machining: A systematic review. J. Manuf. Processes.

[7] Romanowski M., Łukianowicz C., Sutowska M., Zawadka W., Pimenov D.Y., Nadolny K. (2021). Assessment of the Technological Quality of X5CRNI18-10 Steel Parts after Laser and Abrasive Water Jet Cutting Using Synthetic Index of Technological Quality. Materials.

[8] Li H. (2020). Monitoring the abrasive waterjet drilling of Inconel 718 and steel: A comparative study. Int. J. Adv. Manuf. Technol..

[9] Hlavacek P., Hloch S., Nag A., Petru J., Muller M., Hromasova M., Srnicek P. (2020). Effect of rotation direction, traverse speed, and abrasive type during the hydroabrasive disintegration of a rotating Ti6Al4V workpiece. Proc. IMechE Part B J. Eng. Manuf..

[10] Bañon F., Sambruno A., Batista M., Bartolome S., Jorge S. (2020). Study of the surface quality of carbon fiber–reinforced thermoplastic matrix composite (CFRTP) machined by abrasive water jet (AWJM). Int. J. Adv. Manuf. Technol..

[11] Dhanawade A., Kumar S. (2017). Experimental study of delamination and kerf geometry of carbon epoxy composite machined by abrasive water jet. J. Compos. Mater..

[12] Sutowska M., Kapłonek W., Pimenov D.Y., Gupta M.K., Mia M., Sharma S. (2020). Influence of Variable Radius of Cutting Head Trajectory on Quality of Cutting Kerf in the Abrasive Water Jet Process for Soda–Lime Glass. Materials.

[13] Abdullah R., Mahrous A., Barakat A., Zhou Z. (2016). Surface quality of marble machined by abrasive water jet. Cogent. Eng..

[14] Nadolny K., Plichta J., Sutowski P. (2014). Regeneration of grinding wheel active surface using high-pressure hydro-jet. J. Cent. South Univ..

[15] Natarajan Y., Murugesan P.K., Mohan M., Khan S.A.L.A. (2020). Abrasive Water Jet Machining process: A state of art of review. J. Manuf. Process.

[16] Wang Y.F., Yang Z.G. (2008). Finite element model of erosive wear on ductile and brittle materials. Wear.

[17] Bitter J.G. (1963). A study of erosion phenomena: Part II. Wear.

[18] Jegaraj J.J., Babu N.R. (2005). A strategy for efficient and quality cutting of materials with abrasive waterjets considering the variation in orifice and focusing nozzle diameter. Int. J. Mach. Tools Manuf..

[19] Hashish M., Nee A.Y.C. (2015). Waterjet machining process. Handbook of Manufacturing Engineering and Technology.

[20] Karmiris-Obratański P., Kudelski R., Karkalos N.E., Markopoulos A.P. (2020). Determination of the Correlation between Process Parameters and Kerf Characteristics in Abrasive Waterjet Milling of High Strength 7075-T6 Aluminum Alloy. Procedia Manuf..

[21] Dixit N., Sharma V., Kumar P. (2021). Development and characterization of xanthan gum-based abrasive media and performance analysis using abrasive flow machining. J. Manuf. Processes.

[22] Kozhus O., Barsukov G. (2021). The research of the agglomeration process during the formation of an abrasive-polymer compound for waterjet cutting in a fluidized bed installation. Int. J. Adv. Manuf. Technol..

[23] Junkar M., Jurisevic B., Fajdiga M., Grah M. (2006). Finite element analysis of single-particle impact in abrasive water jet machining. Int. J. Impact Eng..

[24] Gostimirovic M., Pucovsky V., Sekulic M., Rodic D., Pejic V. (2019). Evolutionary optimization of jet lag in the abrasive water jet machining. Int. J. Adv. Manuf. Technol..

[25] Zagórski I., Kłonica M., Kulisz M., Łoza K. (2018). Effect of the AWJM method on the machined surface layer of AZ91D magnesium alloy and simulation of roughness parameters using neural networks. Materials.

[26] Arola D., Ramulu M. (1997). Material removal in abrasive waterjet machining of metals surface integrity and texture. Wear.

[27] Srivastava A.K., Nag A., Dixit A.R., Tiwari S., Scucka J., Zelenak M., Hloch S., Hlavacek P. (2017). Surface integrity in tangential turning of hybrid MMC A359/B4C/Al_2_O_3_ by abrasive waterjet. J. Manuf. Processes.

[28] Deaconescu A., Deaconescu T. (2021). Response Surface Methods Used for Optimization of Abrasive Waterjet Machining of the Stainless Steel X2 CrNiMo 17-12-2. Materials.

[29] Phokane T., Gupta K., Gupta M.K. (2018). Investigations on surface roughness and tribology of miniature brass gears manufactured by abrasive water jet machining. Proc. Inst. Mech. Eng. C..

[30] (1997).

[31] (2012).

[32] Stout K.J., Sullivan P.J., Dong W.P., Mainsah E., Luo N., Mathia T., Zahouani H. (1993). The Development of Methods for the Characterization of Roughness in Three Dimensions.

[33] Królczyk G., Kacalak W., Wieczorowski M. (2021). 3D Parametric and Nonparametric Description of Surface Topography in Manufacturing Processes. Materials.

[34] Naser H., Farbod A., Jan K.S., Marcello P. (2015). Effect of entrained air in abrasive waterjet micro-machining: Reduction of channel width and waviness using slurry entrainment. Wear.

[35] Kechagias J., Petropoulos G., Vaxevanidis N. (2012). Application of Taguchi design for quality characterization of abrasive water jet machining of TRIP sheet steels. Int. J. Adv. Manuf. Technol..

[36] Oh T., Cho G. (2016). Rock cutting depth model based on kinetic energy of abrasive waterjet. Rock Mech. Rock Eng..

[37] Xiong J., Wan L., Qian Y., Sun S., Li D., Wu S. (2022). A new strategy for improving the surface quality of Ti6Al4V machined by abrasive water jet: Reverse cutting with variable standoff distances. Int. J. Adv. Manuf. Technol..

[38] Akkurt A. (2010). Cut front geometry characterization in cutting applications of brass with abrasive water jet. J. Mater. Eng. Perform..

[39] Arola D., McCain M.L., Kunaporn S., Ramulu M. (2001). Waterjet and abrasive waterjet surface treatment of titanium: A comparison of surface texture and residual stress. Wear.

[40] Boud F., Murray J.W., Loo L.F., Clare A.T., Kinnell P.K. (2014). Soluble abrasives for waterjet machining. Mater. Manuf. Processes.

[41] Li M., Huang M., Chen Y., Gong P., Yang X. (2019). Effects of processing parameters on kerf characteristics and surface integrity following abrasive waterjet slotting of Ti6Al4V/CFRP stacks. J. Manuf. Processes.

[42] Bukieda P., Lohr K., Meiberg J., Weller B. (2020). Study on the optical quality and strength of glass edges after the grinding and polishing process. Glass Struct. Eng..

[43] Kapłonek W., Nadolny K. (2016). Laser method based on imaging and analysis of scattered light used for assessment of cylindrical surfaces after dynamic burnishing process. Int. J. Surf. Sci. Eng..

